# Potential Therapeutic Interventions Targeting NAD^+^ Metabolism for ALS

**DOI:** 10.3390/cells13171509

**Published:** 2024-09-09

**Authors:** Samuel Lundt, Shinghua Ding

**Affiliations:** 1 Dalton Cardiovascular Research Center (DCRC), Columbia, MO 65203, USA; slhvr@mail.missouri.edu; 2 Department of Chemical and Biomedical Engineering (ChBME), University of Missouri, Columbia, MO 65211, USA

**Keywords:** NAD^+^, NADase, ALS, motor neuron, NMJ

## Abstract

Amyotrophic lateral sclerosis (ALS) is a fatal neurodegenerative disease affecting both upper and lower motor neurons. While there have been many potential factors implicated for ALS development, such as oxidative stress and mitochondrial dysfunction, no exact mechanism has been determined at this time. Nicotinamide adenine dinucleotide (NAD^+^) is one of the most abundant metabolites in mammalian cells and is crucial for a broad range of cellular functions from DNA repair to energy homeostasis. NAD^+^ can be synthesized from three different intracellular pathways, but it is the NAD^+^ salvage pathway that generates the largest proportion of NAD^+^. Impaired NAD^+^ homeostasis has been connected to aging and neurodegenerative disease-related dysfunctions. In ALS mice, NAD^+^ homeostasis is potentially disrupted prior to the appearance of physical symptoms and is significantly reduced in the nervous system at the end stage. Treatments targeting NAD^+^ metabolism, either by administering NAD^+^ precursor metabolites or small molecules that alter NAD^+^-dependent enzyme activity, have shown strong beneficial effects in ALS disease models. Here, we review the therapeutic interventions targeting NAD^+^ metabolism for ALS and their effects on the most prominent pathological aspects of ALS in animal and cell models.

## 1. Introduction

Amyotrophic lateral sclerosis (ALS) is a fatal, adult-onset motor neuron disease typified by the death of motor neurons in the brain, brainstem, and spinal cord. It is a progressive neurodegenerative disease where the initial symptoms are limb/muscle weakness or difficulties swallowing and develops into motor impairments, paralysis, and death [1]. ALS cases are separated into sporadic and familial, which account for about 90% and 10% of cases, respectively. Familial cases can be attributed to a specific genetic mutation, with genes such as *SOD1*, *TARDBP*, *FUS*, or *C9orf72* being the most frequently associated with ALS [2]. Various mechanisms regarding motor neuron degeneration/death, including impairments to mitochondrial function, oxidative stress response, autophagy, and axonal transport, have been observed during ALS [3].

In motor neurons, mitochondria are critical for ATP generation and Ca^2+^ sequestration; however, during ALS, normal Ca^2+^ uptake from the cytosol is slowed, which can induce excitotoxicity. Additionally, the electron transport chain has been found to be damaged in ALS patients, and this can produce inefficient mitochondria with decreased ATP generation but increased reactive oxygen species (ROS) formation [4]. In addition to increased ROS levels, a decreased response to these reactive radicals in neurons may lead to motor neuron death during ALS. Nuclear erythroid 2-related factor 2 (NRF2) binds to the antioxidant response element and regulates expression of nearly 200 anti-inflammatory defense genes. During ALS, NRF2 expression is reduced in motor neurons in post-mortem tissues samples, though whether expression is decreased around disease onset is not known [5]. Altered autophagy has been linked with many of the ALS associated mutations, including to *SOD1* and *TDP-43*, where autophagic machinery is induced early during ALS, but there is an inability to degrade the autophagosomes, leading to proteotoxicity in neurons [6,7,8]. Axonal transport has been found to be disrupted early during ALS and worsens along with disease progression [9,10]. Anterograde and retrograde axonal transport are critical for the trafficking of mitochondria, endosomes, and mRNA transcripts. Loss of transport leads to axon degeneration and death [11]. Unfortunately, most of these mechanisms are linked together, and determining where the initial impairment occurs is challenging.

Nicotinamide adenine dinucleotide (NAD^+^) is a fundamental metabolite in mammalian cells that is critical not only for energy metabolism but also for many cellular responses such as DNA repair, oxidative stress, Ca^2+^ signaling, and the circadian clock [12,13,14]. There are three pathways that can generate NAD^+^: the Preiss–Handler, de novo, and NAD^+^ salvage pathway [15]. The Preiss–Handler pathway has three steps and utilizes nicotinic acid (NA). NA is converted into NA mononucleotide, NA adenine dinucleotide, and then NAD^+^, with these reactions controlled by NA phosphoribosyltransferase, nicotinamide mononucleotide adenylyltransferase (NMNAT1-3), and NAD synthetase, respectively [16]. The Preiss–Handler pathway is expressed strongly in the liver and kidney, with minimal expression in the nervous system [17]. The de novo pathway starts with the amino acid tryptophan and requires nine steps to form NAD^+^, with the last two steps being part of the Preiss–Handler pathway. Expression of the de novo pathway in the nervous system has been reported to be in microglia and astrocytes primarily [18].

**Figure 1:**
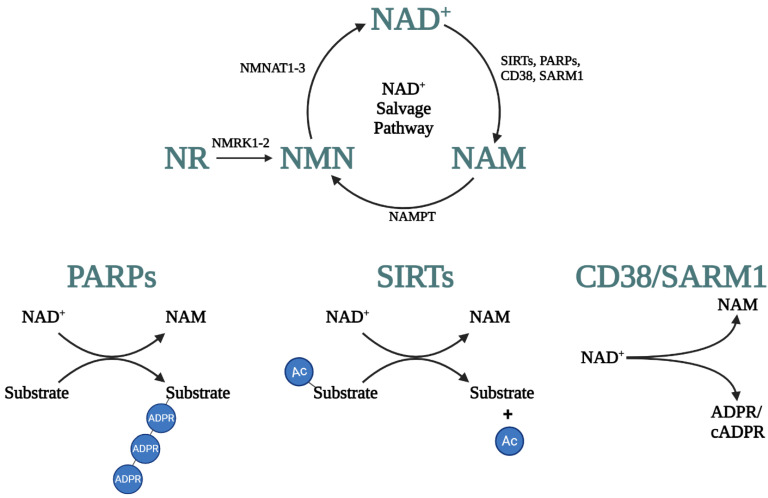
NAD^+^ salvage pathway and NAD^+^-dependent enzymatic reactions. NAD^+^ salvage pathway (**top**) NAM or NR are converted to NMN by NAMPT or NMRK, respectively. NMNAT generates NAD^+^ from NMN. NAD^+^ can be reversibly reduced and oxidized or utilized by NADases (SIRTs, PARPs, CD38, and SARM1), which produce NAM as a byproduct that can be re-used to form NAD^+^. NADase reactions (**bottom**). PARPs add ADPR to substrates. SIRTs remove acetyl groups from target substrates. CD38 and SARM1 generate ADPR/cADPR, which are important for second messenger signaling pathways. All figures were generated using BioRender.com.

The NAD^+^ salvage pathway (Figure 1) is responsible for the majority of NAD^+^ production and begins with nicotinamide (NAM) [19,20]. NAM is converted into nicotinamide mononucleotide (NMN) by nicotinamide phosphoribosyltransferase (NAMPT), which is the rate-limiting step [21]. Nicotinamide riboside (NR) can also produce NMN by nicotinamide riboside kinases (NRK1-2). Next, NMN is transformed into NAD^+^ by NMNAT; NMNATs are part of both the Preiss–Handler and salvage pathways. NAMPT and NMNAT1-3 are expressed in the nervous system, with NAMPT expression restricted to neurons [22,23]. Within neurons, NAMPT is located in the nucleus, cytosol, and mitochondria [24,25]. The NMNATs have different sub-cellular localizations; NMNAT1 is in the nucleus, NMNAT2 in the cytosol/Golgi, and NMNAT3 is in the mitochondria [26]. NAD^+^ can be consumed by NAD^+^-dependent enzymes (SIRTs, PARPs, CD38, and SARM1; Figure 1) and generate NAM as a by-product that can be utilized to regenerate NAD^+^ by the NAD^+^ salvage pathway [22,27]. The maintenance of NAD^+^ levels is crucial for cells, and loss of NAD^+^ homeostasis can be devastating.

The evidence supporting NAD^+^ precursor administration, generally NMN or NR, as a potential therapeutic treatment against aging and disease-related impairments has been well reviewed previously [12,22,28]. However, for ALS in particular, the potential effects of NMN or NR treatments are notable. For many of the factors proposed to cause ALS, augmenting NAD^+^ homeostasis has a positive effect. Autophagy, oxidative stress, mitochondrial dysfunction, and axonal transport have all been found to be positively impacted by improving NAD^+^ metabolism [29,30,31,32]. NR and NMN were able to stimulate mitochondrial autophagy and slow accelerated aging in a Werner syndrome model [33]. NAMPT overexpression or NMN treatment increased cell viability after exposure to H_2_O_2_ [34]. Aged (22–24 months) muscle stem cells treated with NR had mitochondrial respiration and ATP restored to levels observed in muscle stem cells from young (8-month-old) mice [35]. Fast axonal transport is dependent on NMNAT2, and the knockout of NMNAT2 disrupts transport, likely by impairing glycolysis. However, directly adding NAD^+^ resulted in faster anterograde and retrograde transport [36]. As such, while NAD^+^ is highly beneficial to general well-being, it also indicates that ALS pathogenic targets could be especially affected by targeting NAD^+^ metabolism. In this review, we focus on the therapeutic potential of modulators of NAD^+^ metabolism on ALS disease.

## 2. NADase Activity and Cellular Function

There is a well-established relationship between NAD^+^ and health [12,15,37,38]. NAD^+^ levels decline as humans age, suggesting NAD^+^ as a contributing factor to age-related impairments. Lower NAD^+^ levels are not restricted to energy demanding tissues like the brain and skeletal muscle, but they are systemic, with levels reduced in the kidneys, liver, adipose, and plasma as well [39,40,41,42]. The decline in NAD^+^ is due to altered NAD^+^ metabolism (i.e., decreased NAD^+^ biosynthesis and/or increased NAD^+^ degradation). While reduced NAD^+^ production is possible, there is considerable evidence indicating that the activity of NAD^+^-dependent enzymes/NADases (SIRTs, PARPs, CD38, and SARM1) is a significant factor affecting NAD^+^ availability.

SIRTs, i.e., sirtuins, are a family of protein deacetylases important for regulating transcription, the circadian clock, mitochondrial metabolism, and autophagy (Figure 1) [27,38]. There are seven SIRTs that are localized to the nucleus (SIRT1, 6, 7), cytosol (SIRT2), or mitochondria (SIRT3-5) [22]. SIRT activity is beneficial, particularly for neurons during neurodegenerative diseases. SIRT activity has been found to exert protective effects in Alzheimer’s disease (AD), Parkinson’s disease (PD), and ALS [43,44,45,46]. While SIRTs consume NAD^+^, certain protective effects of NAD^+^ are dependent on SIRT activity, which is also called the NAD^+^–SIRT axis. Disruptions to normal NAD^+^–SIRT signaling have been observed in obesity and during aging [47]. Activating the NAD^+^–SIRT axis was found to increase mitochondrial function and biogenesis, which are two of the most common ageing-related impairments [48]. The NAD^+^–SIRT axis is critical for the hypothalamus, which is involved in regulating feeding behavior and circadian rhythmicity. Moreover, the hypothalamus has been proposed as controlling the ageing process, with SIRT activity in certain regions, such as the dorsomedial nucleus and lateral nucleus, mediating age-related impairments [49]. Additionally, following a stroke, the NAD^+^–SIRT axis strongly promoted neurogenesis and differentiation, with the loss of different SIRTs negatively affecting many different pathways [50]. Despite consuming NAD^+^ and reducing NAD^+^ levels, increasing the activity of SIRTs is generally protective, whereas the elevated activity of the other NADases promotes cellular dysfunction. 

PARPs, i.e., poly-ADP-ribosyltransferases, are a critical part of cellular homeostasis. PARPs cleave NAD^+^ and transfer multiple ADP-ribose molecules (poly-(ADP)-ribose/PAR) to target proteins (Figure 1) and are involved with DNA repair and genome maintenance [16,51]. While the PARP family is large, PARP1 accounts for the vast majority of PARP-related activity (~80%) [16,52]. PARPs are also involved in cell death signaling. Excessive addition of PAR polymers (PARylation), results in a form of cell death called parthanatos. In parthanatos, PARP1 becomes overactive, causing PARylation and the movement of apoptosis-inducing factor from the mitochondria to the nucleus, leading to cell death [53]. This increase in PARP activity quickly reduces NAD^+^ levels in cells [22,51,52,54]. While NAD^+^ levels decline with age, PARP activity is potentially elevated, suggesting that PARPs directly contribute to the decrease in NAD^+^ [48]. PARP activity has also been connected to multiple diseases, including muscular dystrophy, ischemic stroke, PD, AD, and ALS [55,56,57,58]. For AD in particular, mutations to PARP1 were found to be associated with altered risk for developing AD in humans [59]. Overall, preventing excessive activation of PARPs is important for cell survival.

CD38 and its homolog CD157 are membrane-bound glycohydrolases. CD38 cleaves NAD^+^ into NAM and cADPR and is important for the regulation of Ca^2+^ signaling (Figure 1) [27,38,60]. There is also evidence that CD38 is the driver of age-related NAD^+^ loss because CD38 expression increases with age [40]. This could be due to the broad effects of CD38 on the nervous system. In the brain, CD38 is likely the primary consumer of NAD^+^, as CD38 knockout mice had a 10-fold increase in brain NAD^+^ levels [61]. These high NAD^+^ levels could potentially be the reason CD38 knockout mice were protected from axon degeneration following cranial nerve axotomy. CD38 knockout mice also experienced reduced microglial activation and immune cell infiltration, suggesting CD38 activity or NAD^+^ availability impact neuroinflammation [62]. CD38 is important for glial cells, especially in neurodegenerative diseases. In astrocytes, CD38 was increased around lesions caused by lysolecithin or multiple sclerosis, and loss of CD38 significantly slowed demyelination [62,63]. The protective effects of CD38 loss may be due to the NAD^+^–SIRT axis. Following retinal damage, CD38 expression increased, and SIRT1 expression decreased. However, the loss of CD38 resulted in increased NAD^+^ and SIRT1 expression while protein acetylation levels, an indicator of SIRT activity, were reduced [64].

SARM1, i.e., sterile alpha and toll/interleukin receptor motif-containing 1, the most recently discovered NADase, is an NAD^+^ hydrolase. It binds to NAD^+^, quickly depletes NAD^+^ levels in axons, and activates axonal degeneration programming (i.e., Wallerian degeneration) [65,66,67]. SARM1 activity is likely dependent on the NMN/NAD^+^ ratio. SARM1 can interact with both NMN and NAD^+^, with NMN stimulating and NAD^+^ inhibiting SARM1 activity [68]. As such, SARM1 and NMNAT activity are strongly connected. Following axon damage, NMNAT expression declines quickly, which increases the NMN/NAD^+^ ratio; however, preventing NMNAT loss can elevate NAD^+^ and inhibit SARM1 activation, thus promoting axon survival [69]. Furthermore, SARM1-dependent axon degeneration is controlled by NMNAT. Reducing NMNAT2 expression either by gene knockout or preventing axonal transport was sufficient to induce widespread axonal degeneration [70,71]. Due to SARM1 controlling an axonal degeneration program, it has been investigated for a role in neurodegenerative diseases. Mouse models of severe nerve injury were protected from neurodegeneration and functional declines when SARM1 was inhibited [72]. In mouse PD neurons, SARM1 activity was potentially elevated, suggesting an involvement in PD pathogenesis [73]. In AD mice, deletion of SARM1 was broadly protective, with reduced neuroinflammation, decreased plaque formation, and improved cognitive performance [74]. While more investigation is necessary, these early findings suggest SARM1 is an important target for potential therapies.

## 3. ALS and NAD^+^ Metabolism

NAD^+^ homeostasis is involved in aging and neurodegenerative diseases, including ALS [15,22,75]. In our recent study using an SOD1^G93A^ ALS mouse model, we found circulating NAD^+^ levels were reduced prior to the appearance of physical symptoms and continued to decline during ALS disease progression [76]. At the end stage, NAD^+^ levels were reduced in the brain and spinal cord as well [76,77,78]. Correspondingly, NAMPT and NMNAT3 expression were also reduced in the spinal cords of SOD1^G93A^ mice [76]. Furthermore, the specific deletion of NAMPT from projection neurons in mice caused a phenotype strikingly reminiscent of ALS, with neuromuscular junction (NMJ) impairments, motor deficiencies, muscle atrophy, paralysis, and eventual death [79,80]. These studies suggest NAD^+^ dyshomeostasis is an aspect of ALS.

The evidence of altered NAD^+^ metabolism extends to ALS in humans. In fact, the potential benefits of NAD^+^ precursors in the de novo pathway and tryptophan metabolism were suggested over two decades ago [81]. More importantly, the NAD^+^ salvage pathway, the dominant biosynthesis pathway, is potentially impaired in ALS patients. In ALS patient blood serum and cerebrospinal fluid (CSF), NAM levels are reduced [82]. The expression levels of NAD^+^-related genes in blood samples could serve as potential biomarkers for ALS, though more investigation is needed [83,84]. Human ALS spinal cord samples have provided strong evidence that NAD^+^ biosynthesis becomes altered during ALS. NMNAT2 mRNA levels are decreased, while overall NAMPT expression appears to be elevated [85]. However, further investigation indicates that intracellular NAMPT, responsible for cellular NAD^+^ generation, is reduced in the spinal cord of ALS patients [79].

NAD^+^ biosynthesis is not the only contributor to impaired NAD^+^ homeostasis in ALS. In human ALS spinal cord samples, nuclear PAR levels were significantly increased in motor neurons. This was observed across different ALS disease subtypes and strongly suggests elevated PARP activity [86]. More recently, SARM1 has been investigated for a role in ALS development. SARM1 mutations may lead to an increased risk for ALS while also negatively affecting disease progression. It was found that human ALS patients have an increased prevalence of harmful gain-of-function SARM1 mutations, which can exert many detrimental effects, the most notable being decreased NAD^+^ levels and increased susceptibility to oxidative stress in neurons [87]. These SARM1 mutants, many of which were determined to be constitutively active, can cause motor dysfunction [88]. While more investigation is needed, this recent evidence strongly indicates that SARM1 mutations, if not a causal factor, are potential risk factors for developing ALS in humans.

## 4. Therapeutic Interventions of ALS by NAD^+^ Precursors

The two most widely investigated NAD^+^ precursors are NR and NMN, both of which can improve NAD^+^ availability and ameliorate many ALS-related detriments (Table 1 and Table 2). Both can delay symptom onset and extend the lifespan of SOD1^G93A^ and FUS^R521C^ ALS mice, though the extent varies, likely due to the dosage and/or starting times of the intervention [76,77,85,89]. Importantly, benefits have been observed across multiple ALS models, with the effects on motor behavior being the most established outcome. This is especially important given the role motor dysfunction has in ALS patients as well as for determining disease progression in ALS mouse models (Table 2). Consistently, across different motor behavior assessments, NMN and NR significantly improved performance. This has been observed in both physically intensive (e.g., accelerated rotarod) and less strenuous (e.g., walking gait) motor assessments [76,77,89]. This delayed motor dysfunction suggests that the motor units (skeletal muscle, NMJ, and motor neuron) in ALS hindlimb muscles strongly benefit from NMN or NR treatments. The evidence from motor unit experiments supports this. For SOD1 and FUS ALS mutations, NR or NMN administration prevented motor neuron death [77,85,89,90,91]. Increasing the activity of the NAD^+^ salvage pathway appears beneficial against ALS. Administering P7C3, an NAMPT-activating molecule, around symptom onset to SOD1^G93A^ ALS mice led to improved motor behavior (rotarod and walking gait) and motor neuron survival [92]. This demonstrates that stimulating the activity of NAD^+^ salvage pathway enzymes can produce similar results as the treatments of the precursor metabolites. In addition, ALS motor neurons provided with NMN or NR displayed increased neurite complexity and out-growth. These gains extended across multiple ALS-related mutations and to human induced-pluripotent stem cell (iPSC)-derived motor neurons [93,94] (Table 1). Observing similar impacts in human motor neurons is important, as it demonstrates that NAD^+^ precursor supplementation is likely translatable to humans.

**Table 1:** In vitro supplementation of NAD^+^ precursors and small molecules.

Model	Cell Type	Treatment	Dose	Duration	Benefits	Ref.
Sporadic	SC-MN	NAD^+^	10 mM	14 days	Increased neurite length	[95]
TDP-43 KD	SC-MN	NAM	0.1 mM	6 days	Axon outgrowth and increased protein synthesis	[96]
SOD1^G93A^	VSC 4.1 cells	Resveratrol	10 µM	2 days	Improved survival; increased ATP, MFN2 and PGC-1α	[97]
SOD1^G93A^	Cortical neurons	Resveratrol	250 nM	2 days	Reduced SOD1^G93A^ toxicity	[98]
Sporadic	hiPSC MN	NAM	0.5 mM	7 days	Improved survival; increased mitochondrial NAD^+^ and respiration; higher CHOP and sXBP1 expression	[99]
		C12	5 µM			
SOD1^G93A^	SC-astrocytes	NR	5 mM	1 day	Increased NAD^+^; decreased astrocyte toxicity; reduced mitochondrial ROS production; higher HMOX1 and SRXN1 expression	[90,91]
		NMN	5 mM			
TDP-43^A315T^	CS-MN	NMN	1 µM	3 days	Increased axon length and neurite intersections and improved mitochondrial ultrastructure	[93]
SOD1^G93A^	SC-MN	NMN	2 mM	2 days	Elevated GSH and NAD^+^, increased neurite length/complexity; improved survival;higher nuclear/cytoplasmic TDP-43 ratio	[94]
SOD1^D90A^	hiPSC MN					
wtTDP-43 OE	SC-MN					

Small molecules: resveratrol, SIRT1 activator; C12, SIRT3 activator. Precursors: NAD^+^, nicotinamide adenine dinucleotide; NAM, nicotinamide; NR, nicotinamide riboside; NMN, nicotinamide mononucleotide. MFN2, mitofusion 2; PGC-1α, peroxisome proliferator-activated receptor gamma coactivator 1-alpha; CHOP, C/EBP homologous protein; sXBP1, spliced X-box binding protein 1; HMOX1, heme oxygenase 1; SRXN1, sulfiredoxin 1.

NMJs are one of the earliest affected regions during ALS. Neurotransmission is altered prior to death of spinal cord motor neurons, while NMJ denervation occurs prior to the development of physical symptoms in SOD1, FUS, and TDP-43 ALS mouse models [100,101]. Fortunately, NMJs and skeletal muscle are among the areas most positively affected by NAD^+^ precursors (Table 2). Innervation of motor endplates was significantly increased, and denervation markers were reduced in SOD1^G93A^ mice treated with NMN or NR [76,85]. NMN also ameliorated motor end-plate alterations (decreased motor endplate area and complexity) and prevented atrophy in skeletal muscle [76]. In addition to these structural changes, the function of the motor unit was improved. In both pre-symptomatic and late-symptomatic SOD1^G93A^ ALS mice, NR strengthened all compound muscle action potential (CMAP) parameters (amplitude, latency, and velocity) [77]. Correspondingly, NMN elevated motor end-plate function. In semitendinosus muscles, evoked end-plate potential responses had higher amplitude and experienced less decline during sustained stimulation; moreover, paired-pulse facilitation indicated that ALS NMJs had increased synaptic plasticity following NMN administration [76]. While improvements to motor axon and NMJ function have been found, how NAD^+^ precursors affect the function and activity of spinal cord motor neurons remains unknown and warrants further investigation.

In the brain, cortical motor neurons become hyperexcitable early during ALS, with enhanced Na^+^ and reduced K^+^ currents. Increased motor evoked potentials, which represent the amount of signaling input into lower motor neurons, has also been reported. Those synapses are glutamatergic and could potentially induce excitotoxicity in spinal cord motor neurons. This results in impaired Ca^2+^ signaling/handling, mitochondrial dysfunction, and excessive reactive oxygen species formation in motor neurons while also inappropriately activating glial cells, all of which are toxic to motor neurons [102]. These mechanisms have been reported in ALS models, including mitochondrial dysfunction, oxidative stress, and neuroinflammation, with NAD^+^ precursors countering many of these impairments (Table 1 and Table 2). Administration of NR or NMN exerts beneficial effects in the nervous system during ALS. NMN and NR can increase many critical metabolites in motor neuron mitochondria, including NAD^+^, ATP, and GSH [77,89,94]. Additionally, NR improved mitochondrial membrane potential, Ca^2+^ handling, and mitophagy, all of which are negatively impacted during ALS progression [77,89]. Mitochondrial alterations were also corrected. In the motor cortex of TDP-43^A315T^ ALS mice, NMN improved mitochondrial ultrastructure, while in the spinal cord motor neurons and skeletal muscle of SOD1^G93A^ mice, mitochondrial morphology and density were increased [76,93]. The benefits of NMN and NR on mitochondrial structure have been demonstrated, but more direct functional analyses of the impact of NAD^+^ precursors on mitochondrial bioenergetics during ALS are still needed.

**Table 2:** In vivo administration of interventions targeting NAD^+^ metabolism.

Model	Route	Treatment	Dose	Duration	Benefits	Ref.
SOD1^G93A^	Sub-cutaneouspump	NAM	7.4 mg/kg BW/day	12 weeks	Longer rotarod retention; lower neurological score; increased NAM in CSF	[82]
SOD1^G93A^	i.p	P7C3	20 mg/kg BW/day	7 weeks	Improved rotarod retention and stride length and reduced MN loss	[92]
SOD1^G93A^	Diet	Resveratrol	160 mg/kg BW/day	8 weeks	Faster treadmill walking speed; increased lifespan and MN survival; higher CMAP and MEP amplitude; decreased p53 acetylation	[103]
SOD1^G93A^	Diet	NR	400 mg/kg BW/day	9 weeks	Improved body weight and grip strength; decreased Cxcl10/Ccl5/Ptgs2/Tnf; reduced Chrna1 and Uchl1	[85]
SOD1^G93A^	Drinking water	NR	400 mg/kg BW/day	10 weeks	Increased Vimentin^+^ and DCX^+^ neurons	[104]
SOD1^G93A^	Oral gavage and diet	NR (w/ and w/o PT/NAC)	185 mg/kg BW/day	14 weeks	Prolonged survival; higher nerve conduction amplitude and velocity; increased NAD^+^; decreased TNF-α/IL2/IL6 and mitochondrial Ca^2+^	[77]
FUS^S57Δ^	Growth media	3-AB	20 µM	9 days	Decreased axonal breaks, slower paralysis onset	[105]
SOD1^G93A^		Olaparib	500 nM			
TDP-43^A315T^		Veliparib	1 µM			
SOD1^G93A^	Oral gavage and diet	NR (w/ PT+Ibu)	185 mg/kg BW/day	14 weeks	Longer rotarod retention and lifespan; improved MN survival; reduced TNF-α/IFNγ/IL1-β in CSF	[89]
FUS^R521C^						
SOD1^G93A^	Diet	NMN	400 mg/kg BW/day	10 weeks	Improved evoked and spontaneous EPP amplitude and increased NMJ plasticity and morphology	[76]

Small molecules: P7C3, NAMPT activator; resveratrol, SIRT1 activator; C12, SIRT3 activator; PT/ pterostilbene, SIRT1 activator; NAC/N-acetyl-cysteine, thiol donor; 3-AB/Olaparib/Veriparib, PARP inhibitors; Ibu/ibudilast, phosphodiesterase inhibitor. Precursors: NAM, nicotinamide; NR, nicotinamide riboside; NMN, nicotinamide mononucleotide; i.p., intraperitoneal injection; MN, motor neuron; Cxcl10, C-X-C motif chemokine ligand 10; Ccl5, C-C motif chemokine ligand 5; Ptgs2, prostaglandin-endoperoxide synthase 2; TNF-α, tumor necrosis factor alpha; IL2, interleukin 2; IL6, interleukin 6; IFNγ, interferon gamma; IL1-β, interleukin 1 beta.

Oxidative stress and neuroinflammation also contribute to ALS pathology. Reactive oxygen species levels are elevated, and the GSSG/GSH ratio, a marker for the oxidative stress response, is increased in the spinal cord and motor cortex of wobbler and TDP-43^A315T^ mice, respectively [78,93]. As mentioned above, NMN and NR can increase GSH availability in motor neurons. Because GSH is the primary antioxidant in cells, higher GSH levels suggest that NAD^+^ precursors can enhance ALS motor neuron resistance to oxidative stress. Supporting this idea, NR treatments improved not only motor neurons’ response to inflammatory cytokine exposure but also the response of astrocytes, microglia, and endothelial cells [89]. NR has a broad impact on neuroinflammation. In the cerebrospinal fluid and lumbar spinal cords of SOD1^G93A^ ALS mice, NR decreased the levels of pro-inflammatory markers and cytokines [77,85]. This stronger response is potentially the result of increased expression of antioxidant response element genes regulated by NRF2, such as SRXN1 and HMOX-1 [91].

While ALS is a motor neuron disease, there are considerable non-cell autonomous effects. Most notably, astrocytes are involved in motor neuron death during ALS. However, both NR and NMN can reduce the toxicity of ALS astrocytes on motor neurons. It appears that this protective effect is being driven by activation of SIRTs [90,91]. In the lumbar spinal cords of SOD1^G93A^ ALS mice, astrogliosis and microgliosis are significantly decreased after NMN or NR treatments, which could be the explanation for higher motor neuron survival [76,77,85,89]. Though the benefits of NMN or NR in isolation are considerable, combining NAD^+^ precursors with other molecules enhances the benefits (Table 2). Treating SOD1^G93A^ ALS mice with NR combined with pterostilbene (PT, SIRT activator), N-acetyl-cysteine (NAC, thiol donor for GSH production), and ibudilast (Ibu, phosphodiesterase inhibitor, which can limit glial cell activation) strengthens the impact of NR. As treatments using only these molecules (i.e., administering PT, NAC, or Ibu without NR) have minimal effects against ALS, these studies suggest NR is responsible for most of the benefits [77,89]. This is why a combination of NR and pterostilbene was used in a human trial for treating ALS. In the trial, patients received 1200 mg EH301 (combination of NR and pterostilbene) daily, with half the dose administered in the morning and half in the afternoon. After 4 months of treatment, patients receiving EH301 had improved ALSFRS-R scores, which measures speech, swallowing, breathing, and motor skills, and patients receiving a placebo had reduced scores. Additionally, the group receiving EH301 had stronger EMG electrical responses after 4 months [106]. Overall, any future treatments involving NAD^+^ precursors should not only investigate these metabolites individually but also investigate molecules that potentially enhance the effects of the NAD^+^ precursors. 

NAM and NAD^+^ have also been investigated for therapeutic effects against ALS, though not to the extent that NMN and NR have been studied. The strongest evidence supporting the therapeutic effect of NAD^+^ was a significant increase in neurite growth of spinal cord neurons in wobbler ALS mice [95]. NAM has received more study than NAD^+^. NAM enhanced neurite and soma morphology while preventing death of human iPSC ALS motor neurons. Additionally, mitochondrial function and NAD^+^ were improved following NAM administration. Interestingly, similar results were obtained by treating iPSC ALS motor neurons with a SIRT3-activating molecule (C12) [99] (Table 1). In SOD1^G93A^ ALS mice, NAM delivered via a subcutaneous osmotic pump demonstrated many benefits. These mice had slower motor decline and extended lifespans. The gene expression profiles in the spinal cords of these SOD1^G93A^ ALS mice were also considerably changed following NAM. Genes relating to mitochondrial structure/function, NAD^+^ homeostasis, and superoxide radical removal were enriched in SOD1^G93A^ mice that received NAM, and a large percentage of the altered genes were regulated by NRF1 [82].

Generally, while NAM treatments have positive effects, NR and NMN are likely more suitable for any potential treatments. Firstly, NAM can function as a SIRT inhibitor and disrupt the NAD^+^–SIRT axis, though the effects of NAM are more nuanced than only reducing SIRT activity [107]. Secondly, NAM incorporation is different from NMN or NR. Exogenous NAM treatments can generate NAD^+^ or be directly methylated into methyl-NAM, while NAM that is generated as a byproduct of NAD^+^ degradation is predominantly reincorporated into NAD^+^ and will not become methylated. This is important because methyl-NAM can be broken down into 4-PY and 2-PY, both of which are potentially toxic [108]. Conversely, NMN can be administered for many months with no apparent harmful side effects [109]. As such, NR and NMN would appear to be the favorable candidates for any future therapies involving NAD^+^ precursor supplementation.

Drugs targeting NADases have also exhibited important benefits against ALS. SIRT activators, in particular, have shown highly promising results. Resveratrol, a SIRT1 activator, has positive effects against ALS symptoms. In ventral spinal cord (VSC) 4.1 cells, a motor neuron–neuroblastoma cell line transduced to express SOD1^G93A^ resveratrol administration improved survival [97]; this effect was also observed when SOD1^G93A^ primary neurons were treated with resveratrol [98] (Table 1). Mitochondrial biogenesis and ATP levels were increased in response to resveratrol both in vitro and in vivo [97]. SOD1^G93A^ mice provided resveratrol had delayed motor symptom onset and extended lifespan, with CMAP amplitude elevated for both upper and lower motor neurons. In the spinal cord, motor neuron number was increased, while gliosis was decreased after resveratrol [103].

PARP inhibitors have also been investigated for treatments against ALS. 3-aminobezamide (3-AB), a non-specific PARP inhibitor, as well as two FDA-approved PARP1/2 inhibitors, Veliparib and Olaparib, were found to slow paralysis and reduce axonal breaks in FUS^S57Δ^, SOD1^G93A^, and TDP-43^A315T^ ALS *C. elegans* worms [105]. In *Drosophila*, inhibition of PARP5 prevented the formation of TDP-43 cytoplasmic inclusions, one of the most prominent pathological hallmarks of ALS disease [110]. In primary rat spinal cord cultures, Veliparib prevented TDP-43-induced neurodegeneration and improved neurite morphology [86]. Importantly, these benefits were observed in different ALS mutants, though in vivo studies in mammals using PARP inhibitors are needed to establish the efficacy of drugs like Veliparib. Also, currently, no studies using inhibitors of CD38 or SARM1 have been performed on any ALS model. Both CD38 and SARM1 are critical NADases, and more investigation into each is needed.

## 5. Future Perspectives

**Figure 2:**
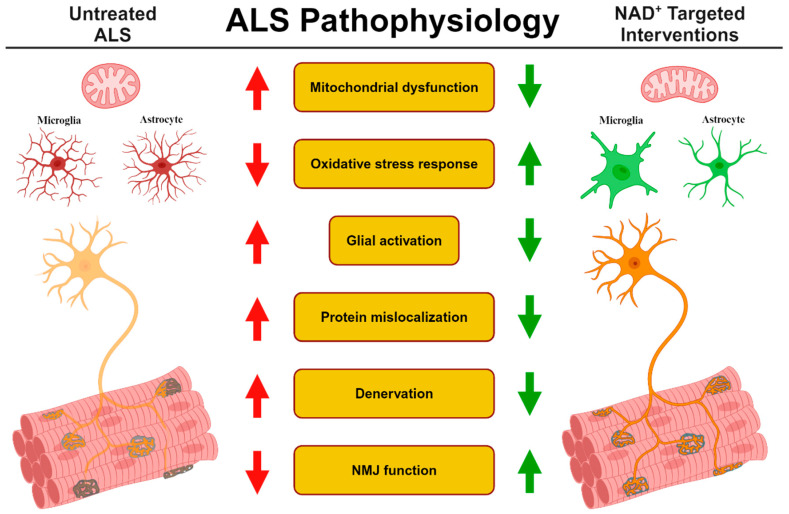
ALS pathophysiology and the effect of targeted interventions involving NAD^+^ metabolism. Treating ALS models with therapeutic interventions altering NAD^+^ metabolism ameliorates many disease-related impairments. Mitochondrial dysfunction, oxidative stress response, activation of glial cells, and protein mislocalization, all of which have been hypothesized as being involved in the development of ALS disease, are corrected from these interventions. Additionally, NMJ innervation and function, sites affected early during ALS development, are improved.

The benefits of targeting NAD^+^ metabolism as a therapeutic strategy against ALS have been demonstrated. Administering NMN or NR can improve motor behavior, reduce oxidative stress, and decrease mitochondrial dysfunction and neuroinflammation, all of which are prominent features of ALS development and pathology. Additionally, the protective effects observed on lower motor units are evident, most notably the increase in motor neuron survival and resiliency of NMJ function and number (Figure 2). These improvements are important and demonstrate that NAD^+^ precursors like NMN or NR should be further investigated to determine whether these effects translate to ALS patients. While NAD^+^ precursors are beneficial, any potential treatments would likely include other metabolites or small molecules. This has already been demonstrated when NR has been paired with a SIRT activator, antioxidant donor, or phosphodiesterase inhibitor.

Furthermore, while the NAD^+^ salvage pathway is responsible for generating the largest proportion of NAD^+^ in cells, other NAD^+^ biosynthetic pathways should be investigated as well. The de novo pathway, which starts with the amino acid tryptophan, has not been as well studied with regards to ALS. The de novo pathway contains more steps than the salvage pathway (nine rather than two), and it is the intermediate molecules generated during these steps that are most interesting. Many of these molecules have demonstrated neurotoxic (3-hydroxykynurenine, quinolinic acid) or neuroprotective (kynurenic acid, picolinic acid) effects [111]. There has been no direct investigation focusing on the role of the de novo pathway and ALS. Analysis of de novo pathway metabolites from ALS patient CSF found that there were no unique changes in ALS patients compared to patients with other neurological diseases [112]. However, whether the de novo pathway increases NAD^+^ production in response to the impairment to the NAD^+^ salvage pathway should be investigated. Targeting the de novo pathway with the purpose of either stimulating NAD^+^ production, enhancing formation of neuroprotective molecules, or limiting formation of neurotoxic intermediates should be studied further.

While the potential benefits of NAD^+^ precursors have been established in animal models, the majority of the investigation has been limited to SOD1^G93A^ ALS mutant mice. However, SOD1 mutations contribute only a small portion of ALS cases, and as such, determining whether targeting NAD^+^ metabolism is just as efficacious in TDP-43, FUS, and C9orf72 ALS disease subtypes is critical. Because ALS is a complex and heterogeneous disease, any potential treatments should be tested on multiple ALS disease sub-types. Additionally, the impact of PARP and CD38 as well as inhibitors of PARP and CD38 on ALS disease needs more investigation. How mammalian models respond to PARP or CD38 inhibition would provide important information for ALS treatments. PARP activity is elevated in ALS spinal cord motor neurons, likely depleting NAD^+^ availability. Additionally, CD38 has be implicated as the primary NAD^+^ consumer in the brain, warranting a more in-depth investigation into a potential role for CD38 in ALS. With NAD^+^ levels declining during ALS progression, combining NMN or NR administration with a PARP/CD38 inhibitor should also be investigated. In summary, the positive effects of NAD^+^ precursors against ALS disease have been shown, and future investigations should focus on the most effective ways of incorporating them into ALS treatment regimens.
